# Post-Transcriptional Modification by Alternative Splicing and Pathogenic Splicing Variants in Cardiovascular Development and Congenital Heart Defects

**DOI:** 10.3390/ijms24021555

**Published:** 2023-01-13

**Authors:** Zubin Mehta, Marlin Touma

**Affiliations:** 1 Neonatal/Congenital Heart Laboratory, David Geffen School of Medicine, University of California Los Angeles, Los Angeles, CA 90095, USA; 2 Department of Pediatrics, David Geffen School of Medicine, University of California Los Angeles, Los Angeles, CA 90095, USA; 3 Children’s Discovery and Innovation Institute, Department of Pediatrics, David Geffen School of Medicine, University of California Los Angeles, Los Angeles, CA 90095, USA; 4 Eli and Edythe Broad Stem Cell Research Center, David Geffen School of Medicine, University of California Los Angeles, Los Angeles, CA 90095, USA

**Keywords:** posttranscriptional modification, alternative splicing, congenital heart defects, transcriptome, splicing variants, genome

## Abstract

Advancements in genomics, bioinformatics, and genome editing have uncovered new dimensions in gene regulation. Post-transcriptional modifications by the alternative splicing of mRNA transcripts are critical regulatory mechanisms of mammalian gene expression. In the heart, there is an expanding interest in elucidating the role of alternative splicing in transcriptome regulation. Substantial efforts were directed toward investigating this process in heart development and failure. However, few studies shed light on alternative splicing products and their dysregulation in congenital heart defects (CHDs). While elegant reports showed the crucial roles of RNA binding proteins (RBPs) in orchestrating splicing transitions during heart development and failure, the impact of RBPs dysregulation or genetic variation on CHDs has not been fully addressed. Herein, we review the current understanding of alternative splicing and RBPs’ roles in heart development and CHDs. Wediscuss the impact of perinatal splicing transition and its dysregulation in CHDs. We further summarize the discoveries made of causal splicing variants in key transcription factors that are implicated in CHDs. An improved understanding of the roles of alternative splicing in heart development and CHDs may potentially inform novel preventive and therapeutic advancements for newborn infants with CHDs.

## 1. Introduction

**Figure 1:**
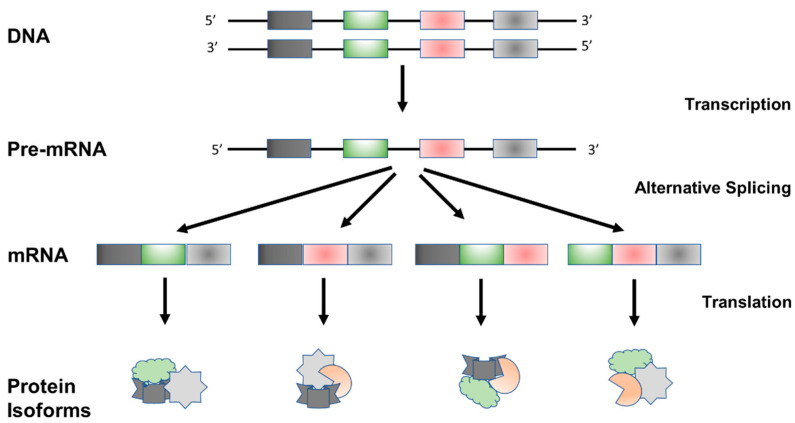
Schematic Representation of Alternative Splicing Process. Alternative splicing leads to functional diversity of transcriptome and proteome by creating a diverse array of protein isoforms from a single gene. Different colors represent difirrent exons and corresponding changes in protein isoforms due to altentaive splicing.

**Figure 2:**
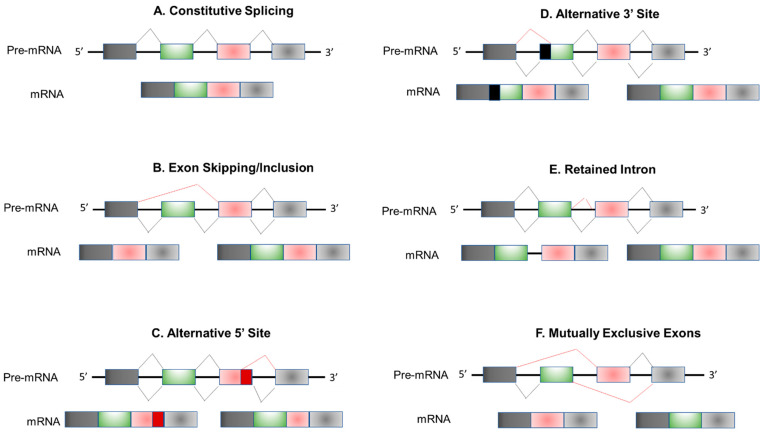
Schematic Representation of Most Common Alternative Splicing Pattern. (**A**) Constitutive splicing. (**B**–**F**) Alternative splicing modes. Skipped exon (**B**), alternative 5′ splice site selection (**C**), alternative 3′ splice site selection (**D**), retained intron (**E**), and mutually exclusive exon (**F**). Different colors represent difirrent exons.

The genome era has introduced new opportunities to understand - novel mechanisms of gene regulation, including post-transcriptional regulation by alternative splicing mechanisms. Orchestrated by the splicing machinery, RNA splicing is a highly regulated post-transcriptional modification process by which introns are removed from nascent pre-mRNAs, leading to the generation of mature mRNAs for translation and protein synthesis [Figure 1]. In contrast to canonical “constitutive” splicing [Figure 2A], alternative splicing exhibits temporal regulation during cellular differentiation, orchestrating tissue homeostasis and organ development by fine-tuning their cellular properties, physiological functions, and developmental trajectories. Meanwhile, dysregulated splicing networks may lead to impaired organ formation and function. Diverse physiological conditions and environmental stimuli may alter splicing decisions leading to the generation of multiple mRNA isoforms from a single gene in tissue-specific and context-dependent manners. This supports the concept that alternative splicing plays crucial roles in proper organ formation and function during the critical stages of mammalian development.

Transcripts from most protein-coding genes in mammals are subject to one or more types of alternative splicing [1,2]. Several alternative splicing types or patterns were described [Figure 2B–F]. Among them, five patterns are most encountered: (1) exon skipping (SE), (2) mutually exclusive exon (MEX) usage, (3) alternative 5′ splice site selection (5′SS), (4) alternative 3′ splice site selection (3′SS), and (5) intron retention (IR) [3].

Remarkably, ES is the most prevalent pattern, in which specific exons, referred to as cassette exons, are either included or skipped from the mature transcript depending on splicing decisions. MEX is less common than ES. In this pattern, one cassette exon is included, while the other is skipped in the mature transcript. The usage of alternative splice start sites or end sites affects the 5′ or 3′ ends, respectively, resulting in shorter or longer forms of exons from the same transcripts. Finally, IR occurs when intronic intervals are retained in the mature transcript, which may be translated or processed by a nonsense-mediated decay mechanism. Alternative splicing reactions are catalyzed by the spliceosome [4]. Spliceosome assembly involves the complex interplay of small nuclear ribonucleoprotein particles (snRNPs, U1, U2, U4/U6, and U5) and other associated proteins. The formation of spliceosomes and their mechanism of action were elegantly investigated and characterized using cryo-electron microscopy studies [5,6,7,8].

Alternative splicing is a ubiquitous process throughout organs, tissues, and cell types. In humans, transcripts of more than 95% of the protein-coding genes are estimated to undergo alternative splicing leading to proteome complexity [9]. In contrast to promoter activity regulation by transcription factors that predominantly affect transcript abundance, alternative splicing events affect mRNAs’ structure and their translation potentials to functional protein isoforms that can exert diverse tissue specificity, cellular localization, binding ability, or enzymatic activity [10]. Furthermore, by altering the reading frame, alternative splicing may affect mRNA localization or translation, leading to protein isoforms of diverse and sometimes opposing functions [11]. The prevalence of alternative splicing has raised questions about its biological importance and functional outcomes. Therefore, establishing the partition of this process in human organ development and disease remains challenging. Indeed, not all splicing products lead to functionally intact protein isoforms at the translational level due to several reasons, amongst them: (1) the splicing event may produce a non-coding transcript lacking a functional open reading frame; (2) the splicing event may lead to a functional non-coding transcript that modulates chromatin accessibility or competes with other RNAs; (3) the splicing event may affect transcript stability leading to antisense mediated decay; (4) the splicing event may alter the subcellular localization of the mature mRNA impairing its translation or function; (5) the nonsense-mediated decay of premature stop codon-containing transcripts; and (6) the splicing events may be overestimated as a result of amplification artifacts.

Alternative splicing events are relatively poorly conserved; however, alternatively spliced exons that exhibit tissue specificity or distinct regulation patterns in response to changes in physiological status or external cues tend to be more conserved at the protein level suggesting putative functional outcomes [12]. Furthermore, large-scale analyses of AS atlases across seven different mammalian species during development suggest that dynamically regulated alternative splicing transitions during organ development tend to be more evolutionarily conserved than the nondynamic, more frequent splicing events [13]. Nevertheless, the extent of alternative splicing and the selection of splicing patterns tend to be different across organs, developmental periods, and types of cassette exons [13].

## 2. Regulation of Alternative Splicing

Operated by the splicing machinery, or the spliceosome, alternative splicing events are driven by various cis-regulatory elements located at the exon-intron boundaries. These splicing enhancer sequences orchestrate splicing decisions by recruiting RNA-binding proteins (RBPs) and other *trans*-acting factors that bind to the RNA molecule and define the exon-intron junctions [14]. Among the most investigated RBPs that contribute to the exon-intron definition are the serine/arginine (SR)-rich proteins (SF2/ASF, SRp20, SRp40, SRp55, and SRp75), the heterogeneous ribonucleoprotein (hnRNP) family of proteins (PTB, hnRNPA1, hnRNP C, hnRNP D, hnRNP E, hnRNP F/H, hnRNP G, and hnRNPH), and the RBPs containing RNA recognition motif (RRM), K homology domain (KH), and zinc-finger domains [15,16].

**Figure 3:**
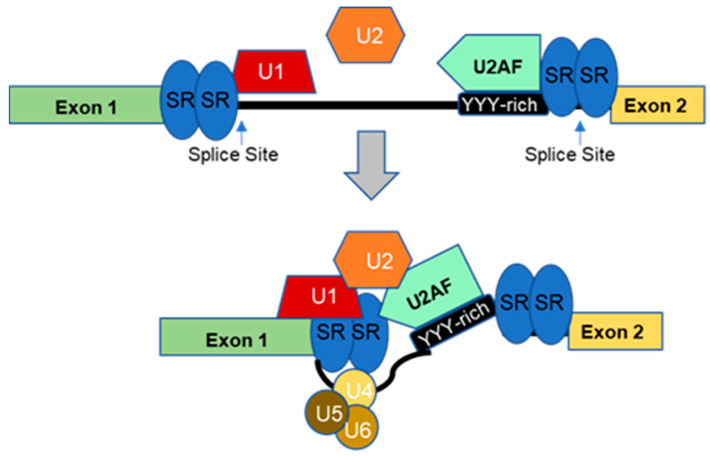
Schematic Illustration of Spliceosome Assembly. Alternative splicing (AS) of a pre-mRNA is carried out by the spliceosome complex. U1 ribonucleoprotein binds to the 5′splice site, while U2AF binds to the 3′splice site and the poly-pyrimidine tract (YYY-rich). RNA-binding proteins (RBPs), such as SR proteins, bind to the splicing recognition motif. U2 ribonucleoprotein mediates the interaction between U1 ribonucleoprotein and U2AF, leading to a conformational change of the RNA-promoting binding of the tri-ribonucleoprotein complex (U4–U5–U6). Modified after Figure 1 in Biamonti et al. (Cells 2020, 9, 34; doi:10.3390/cells9010034) [19]. Copyright lisence: https://creativecommons.org/licenses/by/4.0/.

Upon binding the cis-elements at intron-exon junctions, RBPs promote or repress splice site interactions. Thus, they regulate the splice site selection at an early stage of the spliceosome formation [17,18], although they may contribute to the advanced stages of spliceosome assembly as well [4]. Initially, U1 ribonucleoprotein binds to the 5′splice site (5′ss), while U2AF, a conserved heterodimer that plays a vital role in defining functional 3′ splice sites (3′ss) in pre-mRNA splicing, binds to the 3′ss and the poly-pyrimidine tract (YYY-rich). These early interactions lead to the recruitment of U2 snRNP to the pre-mRNA splice site, followed by the addition of tri-snRNP particles composed of U4/U6 and U5 snRNPs [Figure 3] [19]. Subsequently, RNA helicases facilitate rearrangements of snRNP interactions in the assembled spliceosome, which in turn catalyzes the splicing event [4]. Remarkably, the mutagenesis of minigene reporters demonstrated that splicing efficiency requires cooperative interactions of many RBPs that bind with ‘‘multivalent’’ motifs proximal to alternative exons to carry out the splicing reaction precisely [18,20].

In summary, alternative splicing regulation is carried out by multiple regulatory factors, acting in *cis* or *trans*, to achieve a precise definition of splicing sites. However, to date, the repertoires of proteins that control alternative splicing are not fully characterized. Employing long-read sequencing and examining RNP covalent interactions in post-transcriptional regulation, alongside the current advances in functional genomics and CRISPR-based approaches for modulating splicing, are expected to unfold the complexity of alternative splicing-mediated transcriptome regulation [20,21,22,23,24,25,26]. New sequencing technologies, such as single-cell RNA seq and Nanopore sequencing, are already implemented in neuroscience and cancer biology [27,28,29] and more recently in cardiovascular disease [30], revealing cell-type specific alternative splicing events and their functional impacts on cell behavior and fate. Considering that impaired splicing can lead to various human diseases [31,32,33,34,35,36], efforts tailored to the baseline understanding of tissue-specific and cell-specific alternative splicing processes and their physiologic roles are essential to reveal their contribution to human disease.

## 3. Alternative Splicing Transition during Heart Development

Heart development is a highly dynamic process during which significant transcriptome remodeling occurs in a spatial–temporal regulated manner [37,38]. These changes are driven mainly by transcriptional and post-transcriptional modification mechanisms, including alternative mRNA splicing.

Advancedgenome-wide sequencing and functional genomics tools revealed significant splicing transitions during the differentiation of human embryonic stem cells into cardiac precursors [39,40]. They also uncovered significant differences in alternative splicing patterns between fetal and adult hearts [41,42]. Compared to the adult heart, RI events were found to be more predominant in the fetal heart. Furthermore, cellular proliferation processes were enriched in the fetal-specific alternative splicing events. In contrast, adult-specific events were enriched in energy-related categories [3]. Calcium channel beta2 (CACNB2), tropomyosin 1(TPM1), disabled-1 (Dab1) [43,44], as well as important cell cycle regulators including, pumilio RNA-binding family member 1 (PUM1), calcium/calmodulin-dependent protein kinase 2D (CAMK2D), and anaphase-promoting complex subunit 11 (ANAPC11), exhibit significant splicing differences between fetal and adult hearts [3]. Likewise, sarcomere-related proteins are developmentally regulated via alternative splicing, including cardiac troponin T (cTnT). Exon 5 of cTnT is predominantly expressed in the embryonic heart, encoding a protein domain that increases embryonic cTnT-containing myofilament sensitivity to calcium, as compared to the less sensitive adult cTnT myofilaments, thereby modulating the contractile properties of embryonic myocardium [45,46]. More recently, single-cell RNA sequencing analysis of 996 samples representing the cellular composition of fetal-like (hiPSC-derived cardiac progenitors), healthy adult hearts, and diseased failing hearts further addressed the cellular heterogeneity of fetal and adult hearts [30]. The study also revealed significant reversion of fetal-specific RBPs in the diseased failing hearts that were associated with the re-induction of approximately 1500 fetal-specific isoforms compared to healthy adult hearts [30]. Remarkably, the reactivated fetal-specific isoforms tend to harbor RBP binding sites, have canonical splice site sequences, and contain known upstream polypyrimidine tracts.

Like in prenatal development, alternative splicing transition continues to play an important role as a regulatory component of transcriptomes in the early postnatal development of the murine heart. During this period, dramatic hemodynamic changes occur, driving significant alterations in cellular respiration, metabolism, proliferation, and functional properties. These changes are associated with a highly coordinated alternative splicing program that produces substantial protein isoform transitions that play critical roles in - postnatal heart growth and maturation [47]. Using bulk RNA sequencing, transcriptome dynamics in mouse heart cells, cardiomyocytes, and cardiac fibroblasts, at different prenatal and postnatal stages were recently revealed [48]. While significant splicing changes in cardiomyocytes occur within the first month after birth, indicating an important role of alternative splicing in cardiomyocyte maturation, splicing transitions in cardiac fibroblasts continue beyond the first month. Finally, it is worth noting that alternative splicing products during postnatal heart development are more likely to exert functional consequences when the splicing transition occurs simultaneously in more than one organ, such as splicing events in the heart and brain during development [49,50,51,52,53].

Alternative splicing transitions during heart development are regulated by multiple of RBPs that exhibit significant temporal changes in their expression levels, exerting their functions in cooperative or antagonistic manners [54,55,56]. Out of approximately 1500 RBPs expressed in the heart, 390 cardiac-specific RBPs were identified [54]. Examples of cardiac RBPs that were principally investigated in heart development include *CELF1* (CUGBP Elav-like family member-1), *MBNL1* (muscleblind-like protein-1), *RBFOX1*, *RBFOX2*, *RBM20,* and *RBM24*, among several others [54,55,56,57,58,59]. Studies showed that the MBNL and CELF families play leading roles in the splicing transition during pre- and postnatal heart development [47,49,60]. Like their expression regulation during development, they often lead to reciprocal changes in their shared splicing targets, suggesting antagonistic regulation [49]. While MBNL1 was induced, CELF proteins were suppressed in the postnatal heart. Importantly, both *MBNL1* and *CELF* are regulated by RBM20-mediated alternative splicing during heart development. Correspondingly, *RBM20* loss of function in the adult heart reverts their levels to the embryonic splicing pattern. *Rbfox1* was also identified as a vital regulator for the conserved splicing process of transcription factor *Mef2* family members and was found to be a major player in the reversion of global fetal gene programming in pressure overload heart failure [58].

The identification of the downstream targets of several cardiac RBPs and splicing regulators [Table 1] provided important insights into how these factors can affect heart developmental decisions, physiology, and function [54,55,56,57,58,59,60,61,62]. During the mammalian heart development, RBPs orchestrate the alternative splicing of sarcomere genes that determine the structure and mechanical properties of the heart muscle, best exemplified by the splicing events in *Titin*, which contain the largest number of exons that may be alternatively utilized via splicing regulation, thereby modulating the Titin-based passive tension that determines diastolic ventricular filling. For example, exons 50–219 are shown to be developmentally regulated, with the longer protein isoform (N2BA) predominantly expressed in neonatal hearts, while the shorter protein isoform (N2B) is predominantly expressed in adult hearts [63]. Importantly, this shift toward higher N2B Titin isoform levels increases sarcomere-passive tension and myocardial stiffness. Hence, the relative abundance of the N2BA isoform compared to the N2B isoform determines the myocardium elasticity that controls ventricular filling during diastole [63]. Remarkably, in response to pressure overload, the left ventricles of patients with aortic stenosis exhibit a shift in Titin isoform expression toward the shorter N2B isoform. Consequently, the higher passive tension upon stretch may lead to a decline in cardiac performance [64]. Importantly, the Titin splicing at the PVEK region, which forms the I band with N2BA, is regulated by *RBM20* [63]. Hence, loss-of-function mutations in human *RBM20* have previously been shown to cause hereditary cardiomyopathy due to impaired Titin isoform transition and excessive production of the N2BA isoform in the RBM20-deficient hearts leading to weak Titin filaments and replacement fibrosis [63].

RBM24 is another RBP involved in cardiomyogenesis, and together with RBM20, regulates alternative splicing events of various sarcomere genes, including those encoding myomesin, tropomyosins, *LDB3*, and calcium/calmodulin-regulated kinase II delta (*CaMK IIδ*) [62,65,69,70]. The changes in the isoform expression of these genes were implicated in dilated cardiomyopathy and impaired calcium handling in cardiomyocytes. Furthermore, the cholinergic receptor muscarinic 2 (*CHRM2*) was identified as a target for RBM24, revealing a new mechanism by which *RBM24* variants may modulate cardiac conduction and contractility [65,70]. SRp38 was also found to regulate cardiomyocyte contractility. The loss of *SRp38* in mice disturbed the splicing of triadin, a protein that controls calcium release from the sarcoplasmic reticulum during excitation–contraction (E–C) coupling [71]. Likewise, cardiomyocyte-specific ablation of SF2/ASF resulted in the impaired postnatal splicing switch of *CAMK IIδ,* leading to a defect in the E-C coupling, dilated cardiomyopathy, and heart failure [72]. More recently, Quaking (QKI), an hnRNP protein [35], was identified as a critical alternative splicing regulator in cardiomyocyte differentiation and maturation that is required for heart development and function.

## 4. Dysregulated Alternative Splicing in Congenital Heart Defects (CHDs)

### 4.1. Splicing Transition in CHDs

Only recently has the role of alternative splicing in CHDs gained further attention. Global transcriptome profiling studies helped examine alternative splicing partitions in different CHD phenotypes. In the following, we highlight some representative studies and reference the other equally important literature [Table 2].

Bicuspid aortic valve (BAV) is a common CHD affecting 0.5–2.0% of the general population and associated with risks for aortic dilatation and dissection. Using Affymetrix exon arrays, Fibronectin (FN) splicing isoforms were analyzed in dilated and nondilated ascending aorta from human BAV samples (n-69) and normal tricuspid aortic valve (TAV) samples *(n* = 40). An alternatively spliced extra domain A of FN (EDA-FN), which is essential for tissue repair, was found to be correlated with the maximum diameter of TAV but did not increase in dilated aorta tissues from BAV [73]. Remarkably, transforming growth factor-β (TGFβ) treatment increased EDA-FN isoform expression in the cultured cells derived from TAV patients but not in the cells derived from BAV patients. Together, this indicates that differences in the TGFβ signaling pathway may explain the impaired inclusion of EDA-FN in BAV patients.

Hypoplastic left heart syndrome (HLHS) is a serious and complex form of CHD, characterized by left ventricle hypoplasia and single ventricle physiology, with compensatory hypertrophy and hemodynamic overload of the right ventricle (RV). Using a genome-wide Affymetrix exon array, an extensive transcriptome analysis, including characterizing alternative splicing profiles, of the RV of six HLHS patients and the RV and left ventricle (LV) from control subjects, revealed distinct differential gene expressions and alternative splicing events in the RV myocardium of the HLHS patients compared to the RV and LV from the control heart, representing a unique molecular signature of HLHS, involving 180 differentially expressed genes and 1800 differentially spliced transcripts that were enriched in cell metabolism, cytoskeleton, and cell adhesion. Furthermore, some dysregulated genes can be quantified in plasma samples and serve as molecular biomarkers for prognostication classification. Such examples include those involved in calcium transporters (*SLC8A1* and *CACNB2*) and energy production (*COX4l1* and *ATP4A1*) as well as secreted factors (*IFI44* and *VEGFA*) [74].

Tetralogy of Fallot (TOF) is the most common cyanotic CHD phenotype that is typically associated with significant remodeling of the RV outflow tract (RVOT) due to pulmonary stenosis. By characterizing alternative splicing in RVOT specimens obtained from TOF patients, the small cajal body-specific RNA 1 (scaRNA1) was found to be downregulated-. Mechanistically, scaRNA1 loss in the primary cultured cells derived from the right ventricle of TOF patients dysregulated the splicing of important regulators of heart development, including *GATA4*, *NOTCH2*, *DICER1*, *MBNL1*, and *MBNL2*. themis-splicing of the cardiac development genes was also observed in Zebrafish in response to the morpholino-mediated silencing of scaRNA1 and was associated with decreased pseudouridylation in Spliceosomal RNA U2, potentially leading to reduced communication between the first and second heart fields and conotruncal misalignment, the hallmark in TOF [75].

Ventricular noncompaction is characterized by abnormal ventricular trabeculation and progressive cardiac dysfunction. The genetic cause of this disorder remains elusive. RNA-binding protein with multiple splicing (Rbpms) is highly expressed in the heart and contains a conserved RNA recognition motif (RRM) [76]. A recent report [66] demonstrated that genetic deletion of a novel Rbp with multiple slicing (Rbpms) leads to early lethality in neonatal mice caused by CHDs. Mechanistically, the Rbpms-depleted cardiomyocytes undergo an early exit from the cell cycle. This cytokinesis defect was also observed in human iPSCs-derived cardiomyocytes that carried the *RBPMS* gene deletion and were found to be associated with dysregulated RNA splicing of genes enriched in cytoskeletal signaling pathways, including the cardiac enriched LIM domain protein 5 (Pdlim5). Specifically, the Rbpms loss resulted in a substantial increase in Pdlim5-short isoforms that impaired cardiomyocyte division and resulted in premature binucleation, leading to ventricular noncompaction.

DiGeorge syndrome results from a micro-deletion located on the short arm of chromosome 22, which includes the *HIRA* gene and is commonly associated with TOF and other CHDs. It was shown that an intronic sequence (22k48) transcribed by the *HIRA* opposite strand, but not translated, undergoes alternative splicing [77]. Importantly, the haploinsufficiency of this intronic sequence may lead to the stigmata of DiGeorge syndrome, including CHDs, indicating that the non-coding intron retention mechanism of splicing can potentially exert pathogenic impacts on CHDs.

### 4.2. Role of Pathogenic Variants in RBPs in CHDs 

Evidence from the human genetics and mouse models implicated pathogenic variants in RBPs in CHDs by impairing the splicing of their target genes [Table 2]. For example, the pathogenic variants in *RBM10*, a ubiquity expressed RBP, are known to cause TARP(Talipes equinovarus, Atrial septal defect, Robin sequence, and Persistent left superior vena cava) syndrome, an X-linked disorder that affects males [78,79,80]. Pathogenic variants in *RBFOX2* were implicated in HLHS [81]. Furthermore, *RBFOX2* may contribute to transcriptome dysregulation in RVs from HLHS patients [67]. Indeed, it was demonstrated that most of the transcripts that are differentially regulated in HLHS compared to control are targets for *RBFOX2* with 3′UTR binding sites contributing to mis-splicing [67]. Moreover, conditional deletion of *Rbfox2* in mouse embryos led to perturbation of yolk sac angiogenesis and complex heart defects recapitulating several features of HLHS. Remarkably, *Rbfox2* mutant heart-derived transcriptomes analysis identified dysregulated alternative splicing affecting extracellular matrix (ECM) and cellular adhesion networks. This was found to be mediated by Rho GTPases, two of which were identified as targets for *Rbfox2* [59]. By affecting the splicing of their downstream sarcomere gene products, such as Titin, pathogenic *RBM20* variants can cause human arrhythmogenic dilated cardiomyopathies (DCMs) and sudden cardiac death by disrupting the Ca^2+^ handling [63,68,82]. However, no disease-associated *RBM24* variants have been described to date.

Among the key core transcription factors in heart development is *TBX5*, a T-box transcription factor that is required for heart and limb morphogenesis. Pathogenic variants in *TBX5* are a known cause of Holt–Oram syndrome featured by CHDs and forelimb maldevelopment. Other than the known role of TBX5 in transcription regulation, it was also demonstrated that TBX5 plays a role in pre-mRNA splicing via the forming of a complex with the splicing factor SC35, an SR protein that was shown to be an essential splicing factor involved in spliceosome assembly and a regulator of alternative splicing [68]. Specifically, TBX5 acted as an RBP with high specificity to the 5′ss of the *ANF* minigene but not to the 3′ss. Intriguingly, *TBX5* overexpression improved the splice site definition and enhanced the splicing efficiency of *ANF* mediated by SC35. Moreover, the pathogenic variant (G80R) that affects TBX5 splicing performance is directly linked to the pathogenesis of Holt–Oram syndrome and featured with complete penetrance of CHDs due to significant mis-splicing of mRNA. In contrast, other variants that do not affect TBX5 splicing function usually have incomplete penetrance in CHDs [83].

### 4.3. Role of Splicing Site Variants in CHDs

Advances in genomics platforms improved the detection of rare pathogenic splicing genomic variants that alter canonical splice sites, thus resulting in splicing and functional defects of their genes or impacting their promoter activity and regulatory elements. Nonetheless, establishing the causal roles of these variants and identifying their downstream targets in CHDs remain challenging. Few reports described pathogenic splicing variants in key cardiac transcription regulators or structural genes leading to developmental perturbation and CHDs [Table 3]. *GATA4*, which encodes GATA Binding Protein 4, is a key transcription factor that plays an important role in cardiac development and was implicated in CHDs. By performing analysis of the *GATA4* variant in an Indian patients with CHDs, two intronic splice site variants ([g.83271C>A/M] and [g.86268A>R]) were predicted to affect intronic splice sites at the enhancer and silencer motifs based on in silico prediction analysis, indicating that the non-coding pathogenic splicing variant can introduce splicing defects that lead to CHDs [84].

Othersplicing variants of key regulatory genes may contribute to CHDs. One example is the Regulator of Calcineurin 1 (*RCAN1*), which was linked to CHDs associated with Down syndrome. To examine if *RCAN1* contributes to non-syndromic cases of CHDs, *RCAN1* exons and flanking regions from 128 patients with non-syndromic CHD and 150 normal controls were sequenced, leading to the detection of six novel heterozygous variants in the *RCAN1* gene in CHD cases, although they were absent in control cases. In particular, the g.482G>T variant was found to enhance *RCAN1* promoter activity leading to overexpression of the *RCAN1.4* isoform, potentially causing CHDs in the absence of Down syndrome [85].

Pathogenic splicing variants in genes encoding structural proteins were also implicated in CHDs. Encoded by *FBN1*, Fibrillin 1 is a key constituent of ECM. Pathogenic *FBN1* variants are linked to Marfan’s syndrome (MFS) and mitral valve–aorta–skeleton–skin (MASS) syndrome. However, *FBN1* variants are mostly enriched in intronic sequences, making the prediction of their pathogenicity and establishing the genotype-phenotype correlations very challenging. A recent study [86] reported two *FBN1* deep intronic variants ([c.6872-24T>A] and [c.7571-12T>A]) in two unrelated patients who were affected with MFS/aortic disease and MASS syndrome, respectively. Remarkably, both variants led to the retention of intronic regions, resulting in changes in the reading frame and the introduction of premature stop codons. The pathogenic variants of RBPs and the splice site variants in CHDs are summarized in [Table 3].

### 4.4. Splicing Variants Leading to Congenital Conduction Defects (Arrhythmias)

Pathogenic splicing variants may affect ion channel assembly and function, leading to developmental defects in conduction. It has been shown that the potassium channel (Kv11.1) isoform switch represents a novel mechanism of the congenital long-QT syndrome. A novel splice site variant in *KCNH2*, which encodes Kv11.1, was detected in an expanded family, affecting the relative abundance of the full-length Kv11.1a isoform and the truncated Kv11.1a-USO isoform. This, in turn, was dictated by the competition between *KCNH2* alternative splicing and the alternative polyadenylation mechanisms [87]. Splicing defects may also impair voltage-gated sodium channels. A recent report demonstrated that a non-muscle isoform of *RBFOX2* [RBFOX2_40_] is upregulated in heart tissue from myotonic dystrophy 1 (DM1) patients leading to elevated *CELF1* and a global miRNA suppression [23]. By modeling in mice, *Rbfox2_40_* isoform overexpression caused a mis-splicing of the voltage-gated sodium channel transcripts, creating a pro-arrhythmic status that altered the channel electrical properties leading to conduction defects.

Arrhythmogenic right ventricle dysplasia (ARVD) is a rare inherited disorder that involves the replacement of RV cardiomyocytes with fibro adipose tissues that consequently leads to ventricular arrhythmias. ARVD cases with dominant inheritance and incomplete penetrance are caused by heterozygous *PKP2* mutations. Interestingly, the first reported ARVD case with recessive inheritance was caused by a homozygous cryptic splice variant in *PKP2* (c.2484C>T), which was initially annotated as a synonymous variant [88]. However, further analysis of the proband’s mRNA uncovered the disruption of the *PKP2* reading frame and alteration of *PKP2* splicing outcomes caused by this cryptic splice site variant leading.

## 5. Conclusions

Alternative splicing is a ubiquitous process that plays important roles in transcriptome regulation and proteome diversity. The current literature evidence supports the important regulatory roles of alternative splicing in cardiovascular development and CHDs. Splicing transition is controlled by a complex and intricate network of RBPs, which orchestrate the splicing transition of their targets during heart development and can be dysregulated in CHDs. Pathogenic variants of RBPs may alter the splicing decisions of their targets and account for substantial developmental perturbation leading to CHDs. Pathogenic splicing variants of key core cardiac transcription factors and structural genes can be causal to CHDs. 

Taking into consideration the existing challenges in establishing the partition of his vital process in human heart development and disease, massive efforts tailored to a comprehensive baseline understanding of tissue-specific and cell-specific alternative splicing transitions and their physiologic roles during heart development are essential. Utilizing cutting-edge sequencing technology, such as single-cell and long-read RNA sequencing; examining RNP covalent interactions in post-transcriptional gene regulation, and employing functional genomics and CRISPR-based approaches for modulating splicing are expected to unfold the complexity of alternative splicing-mediated transcriptome regulation mechanisms at the cell-type specific level and reveal their functional impacts on cell behavior and fate during development and their contributions to human CHDs. Multilayered collaborative bioinformatics, functional genomics, and mechanistic approaches for examining RBPs dysregulation and elucidating the causal impact of newly discovered splicing variants in CHDs are critical to uncover new mechanisms and pave the way to novel diagnostic and targeted approaches for infants with CHDs. 

## Figures and Tables

**Table 1:** RNA binding proteins and their main targets in heart development.

RNA Binding Protein	Main Target(s)	CHD/Condition	References
RBFOX1	*MEF2*	Heart Failure/Fetal-like program	[44]
RBM24	*LDB4, CAMKII* *δ* *, TPM, MyoM*	Sarcomerogenesis	[51,54,55,56]
RBM20	*TTN*	Ventricular Elasticity	[52,53]
CELF1	Fetal-like program	Heart Development/ Myofibrinogenesis	[57,64,65]
MBNL1	Fetal-like Program	Heart Development	[57,64,65]
SRp38	Triadin	Excitation-Contraction Coupling	[62]
ASF/SF2	*CAMKII* *δ*	Excitation-Contraction Coupling	[63]
RBpms	*Pdlim5*	Left Ventricle Noncompaction	[66]
RBFOX2	*Rho GTPases*	Hypoplastic Left Ventricle	[67]
TBX5/SC35	*RNF*	Holt-Oram Syndrome	[68]

**Table 2:** Summary of Topics Highlighted in the Review. TOP cardiac-enriched genes encoding RNA binding proteins and other cardiac genes that are affected by alternative splicing or splicing variants are listed based on their functional categories.

Alternative Splicing in Congenital Heart Disease
**RNA Binding Protein Genes**
*RBM10, RBM20, RBM24, RBFOX1, RBFOX2, SC35, SFB31, ASF/SF2, RBFOX2,* *RBpms, PUM1, CELF1, MBNL1, SRp38*
**Splice Variants/Cardiac Transcription Regulators**
*TBX5, GATA4, HAY2 RCAN1*
**Splice Variants/Cardiac Conduction Genes**
*PKP2, KV11.1*
**Cardiac Structure/Sarcomere Genes**
*FBN1, TTN, TNNT2, TPM1, MyoM, LDB3*

**Table 3:** Genes affected by pathogenic splicing variants and their contribution to CHDs. Notes: TARP: Talipes equinovarus, Atrial septal defect, Robin sequence, and Persistent left superior vena cava; ARVD: Arrhythmogenic Right Ventricle Dysplasia; AVSD: Atrial Ventricular Septal Defect.

Reference Citation	Affected Gene	Variant	Phenotypes
[63] Guo et al. *Nat Med* (2012) 18 (5), 766-773	*RBM20*	S635A	Dilated Cardiomyopathy
[78] Wang Y et al. *EMBO Mol Med* (2013) 5,1431-1442	*RBM10*	Del of 1292nucleotides (ChrX: 46929367-46930658 bp)	TARP Syndrome/CHD
[79] Tessier et al. *BMB Research Notes* (2015) 8,46	*RBM10*	Tandem donor splice site (GTGGTG) in *RBM10* exon 10	TARP Syndrome/CHD
[80] Johnston JJ et al. *The American Journal of Human Genetics* (2010) 86,743-748	*RBM10*	c.1235G>A; c.1893_1894insA	TARP Syndrome/CHD
[82] Beqqali A et al. *Cardiovascular Research* (2016) 112, 452-463	*RBM20*	c.2737G>A	Dilated Cardiomyopathy
[68] van den Hoogenhof et al. *Circulation* (2018) 138, 1330–1342.	*RBM20*	Multiple Variants	Dilated Cardiomyopathy
[83] Fan C et al. *The Journal of Biological Chemistry* (2009) 284, 38, 25653-25663	*TBX5/SC35*	G80R	HOLT-Oram Syndrome/CHD
[84] Bose D. et al. *Mutat Res Fund* (2017) 803-805, 26-34	*GATA4*	g.83271C>A/M (intronic variant); g.86268A>R (intronic Variant)	Nonsyndromic ASD/VSD/AVSD
[85] Li X et al. *Pediatr Cardiol* (2018) 39, 226-235	*RCAN1*	g.482G>T (intronic variant)	Nonsyndromic ASD/VSD/AVSD
[86] Fusco C et al. *Genes* (2019) 10 (6), 442	*FBN1*	c.6872-24T>A; c. 7571-12T>A	Marfan’s Syndrome/CHD
[87] Gong Q et al. *Circ Cardiovascular Gent* (2014) 7 (4), 482-490	*KCNH2*	IVS9-2delA (a deletion of the A in AG dinucleotide of the 3’ acceptor site of *KCNH2* intron 9)	Long QT Syndrome
[88] Awad MM et al. *Human Mutat* (2006) 27 (11), 1157.	*PKP2*	c. 2484C>T + c.2484C>T	ARVD

## Data Availability

Not applicable.

## Abbreviations

ARVD	Arrhythmogenic Right Ventricular Dysplasia
BAV	Bicuspid Aortic Valve
CHDs	Congenital Heart Defects
E–C	Excitation–Contraction
HLHS	Hypoplastic Left Heart Syndrome
LVNC	Left Ventricular Noncompaction
RBPs	RNA-Binding Proteins
RBPMS	RNA-Binding Protein with Multiple Splicing
RVOT	Right Ventricular Outflow Tract
TAV	Tricuspid Aortic Valve
TOF	Tetralogy of Fallot
